# Cell-Targeted Inhibition of CaMK4 Suppresses Tertiary Lymphoid-like Structure Development in Lupus-Prone Mice

**DOI:** 10.3390/ijms27073190

**Published:** 2026-03-31

**Authors:** Simin Jamaly, Mehrdad Rakaee, Kunihiro Ichinose, Kayaho Maeda, Kotaro Otomo, Tomohiro Koga, Maria G. Tsokos, George C. Tsokos

**Affiliations:** 1 Department of Rheumatology, Oslo University Hospital, Rikshospitalet, 0372 Oslo, Norway; 2 Department of Medicine, Beth Israel Deaconess Medical Center, Harvard Medical School, Boston, MA 02215, USAotomo122@gmail.com (K.O.);; 3 Department of Cancer Genetics, Oslo University Hospital, Radiumhospitalet, 0310 Oslo, Norway

**Keywords:** autoimmunity, CamK4, lupus, nephritis, tertiary lymphoid structure

## Abstract

Current treatment of lupus nephritis (LN) relies on broad immunosuppression and often fails to eradicate intrarenal immune niches that sustain inflammation. Tertiary lymphoid structures (TLS)—organized aggregates of immune cells forming in chronically inflamed non-lymphoid tissues—are increasingly recognized as drivers of local immune activation and tissue injury in LN. We previously showed that genetic CaMK4 deficiency suppresses autoimmunity and nephritis in lupus-prone mice. Here, we tested whether CaMK4 regulates renal TLS-like organization. Using kidneys from MRL/*lpr* mice that were CaMK4-deficient or treated with KN93-loaded nanoparticles targeted to CD4^+^ T cells or podocytes (anti-podocin), we compared findings with vehicle-treated controls. TLS-associated inflammation and maturation were quantified by mean fluorescence intensity of CD3, CD20, Ki67, and α-SMA. Across genetic and targeted-treatment arms, CaMK4 inhibition reduced all assessed markers, with uniform suppression of CD20 signal, highlighting a key role for B cells in TLS maintenance. Notably, podocyte-targeted KN93 most strongly suppressed TLS-like formation, implicating podocyte-driven pathways in interstitial inflammation and lymphoid neogenesis through previously underappreciated mechanisms. These data identify CaMK4 as a regulator of TLS-like architecture in LN and support the translational potential of cell-targeted CaMK4 inhibition to disrupt local immune recruitment while limiting systemic toxicity.

## 1. Introduction

Lupus nephritis (LN), affecting approximately 20–60% of patients with systemic lupus erythematosus (SLE), is a major cause of disease-related morbidity and mortality, leading to end-stage kidney disease in 10–20% despite immunosuppressive therapy [1,2]. Although therapeutic options have expanded, more than 40% of patients still fail to achieve complete renal remission, and treatments carry substantial toxicity [3]. Consequently, many patients have persistent renal inflammation and remain at high risk for kidney failure [4].

A likely reason for this therapeutic gap is that systemic immunosuppression fails to eradicate local renal immune niches. Chronic inflammation promotes the formation of renal tertiary lymphoid structures (TLS) with organized T- and B-cell zones, follicular dendritic cells, and high endothelial venules, which perpetuate local autoantigen presentation and autoantibody production [5]. In LN, TLS presence and maturity correlate with more aggressive histology and worse outcomes [6], implying that these ectopic lymphoid sites orchestrate cycles of in situ immune activation that systemic therapies only partially suppress.

Recognition of TLS as local amplifiers of renal autoimmunity underscores their role as central regulators of immune and kidney-resident cells. Calcium/calmodulin-dependent protein kinase IV (CaMK4) is upregulated in both compartments in LN [7]. In T lymphocytes, heightened CaMK4 activity promotes Th17 skewing, disrupts regulatory T-cell (Treg) function, and amplifies systemic autoimmunity [8]; in podocytes, it drives cytoskeletal remodeling and injury, contributing to proteinuria [9,10]. In MRL/*lpr* mice, CaMK4 deletion attenuates autoantibody titers, diminishes systemic immune activation, and reduces glomerular inflammation and injury, establishing CaMK4 as a dual immune–renal therapeutic target in LN [11].

Using nanoparticle platforms for cell-type-specific suppression of CaMK4 signaling in preclinical models, we found that CD4^+^ T-cell-targeted inhibition preserved glomerular architecture, curtailed inflammatory cytokines, and limited the emergence of autoantibody-producing niches [12], whereas podocyte-targeted inhibition mitigated LN without affecting systemic autoimmunity [9].

What remained unclear was whether CaMK4 exerts its pathogenic effects predominantly through immune cells, podocytes, or a cooperative interaction between the two. To address this, we examined kidneys from MRL/*lpr* mice with global CaMK4 deficiency and from mice treated with CD4- or podocin-targeted KN93 nanogels. TLS were quantified by CD3, CD20, Ki-67, and α-SMA staining, and with semi-quantitative scoring of TLS presence and size. Notably, podocyte-specific CaMK4 inhibition disrupted TLS formation and maturation, thereby reducing inflammatory activity in LN. These findings support a mechanistic link in which CaMK4 activity within podocytes promotes injury and fosters TLS-driven renal inflammation.

## 2. Results

### 2.1. Renal Histopathology and TLS Distribution

**Figure 1:**
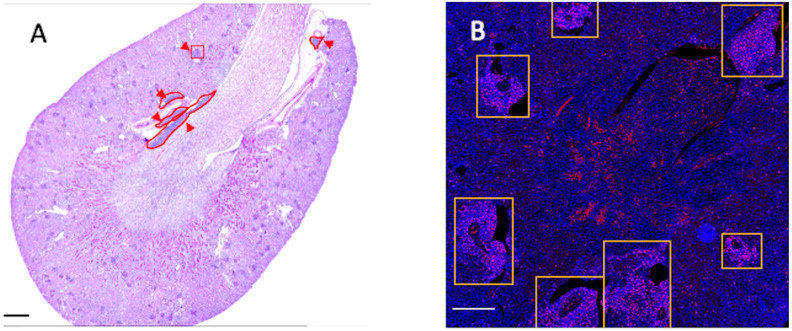
Representative whole kidney sections from MRL/*lpr* mice. (**A**) Periodic acid–Schiff (PAS) staining (arrows indicate the regions shown at higher magnification) and (**B**) immunofluorescence staining for Ki-67 show areas indicated by boxes corresponding to tertiary lymphoid structures (TLS). Scale bars (**A**) = 100 mm, (**B**) = 75 mm.

**Figure 2:**
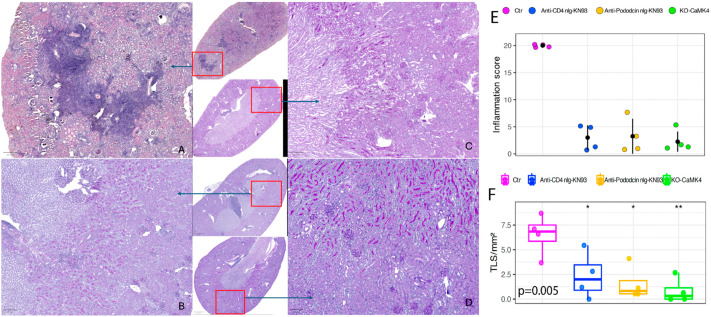
PAS-stained kidney sections, inflammation scores, and tertiary lymphoid-like structure density in MRL/*lpr* mice. (**A**–**D**) Representative PAS-stained sections and Banff Classification System (BCS = 0–3) inflammation scores. (**A**) Control mice (BCS = 3; Inflammation Score = 80%). (**B**) Mice treated with KN93-loaded and CD4-tagged nanogels (BCS = 0; Inflammation Score < 5%) (**C**) Camk4 knockout (KO) mice (BCS = 0; Inflammation Score ≤ 1%). (**D**) Mice treated with KN93-loaded and podocin-tagged nanogels (BCS = 0 Inflammation Score 1%). Red boxes indicate the selected areas, and arrows indicate the regions shown at higher magnification. Scale bar = 20 µm for magnified view of the indicated region and 100 µm for whole kidney image. (**E**) Quantification of mean inflammation scores per groups showing substantial reduction in all CaMK4-targeted groups compared to controls. Individual mice are shown as colored dots, and the group mean is indicated by a black dot. (n = 4 mice per group). (**F**) Quantification of tertiary lymphoid-like structure density in kidney sections, expressed as TLS per mm^2^. Each dot represents one mouse; boxes indicate median and interquartile range (whiskers = min–max). TLS density was significantly reduced in all CaMK4-inhibited/deficient groups compared with vehicle controls (*p* = 0.005; * *p* < 0.05, ** *p* < 0.01) (n = 4 mice per group).

Control MRL/*lpr* kidneys showed classic LN pathology, including interstitial fibrosis, tubular atrophy, and diffuse mononuclear infiltrates in cortex and medulla. TLS were defined and quantified as shown in Figure 1. TLS aggregates were localized predominantly in the medulla near large blood and lymphatic vessels, with fewer in the cortex (Figure 2A). Genetic CaMK4 deficiency and CD4^+^ T-cell- or podocyte-targeted KN93 delivery significantly reduced Banff inflammation scores (Figure 2B–E), However, the reductions did not differ significantly between the three treatment arms, indicating that this general inflammation biomarker does not discriminate treatment-specific effects. TLS density (TLS/mm^2^, min–max) was highest in control MRL/*lpr* mice at 6.34 (3.68–8.70), followed by the CD4-targeted KN93 group at 2.53 (0–5.86) and the podocyte-targeted KN93 group at 1.43 (0.53–4.11). The lowest density was observed in CaMK4-deficient mice, with a mean of 0.88 (0–2.67). No significant differences were detected among the three treatment arms (Figure 2F).

### 2.2. T and B Cell Content Within TLS

**Figure 3:**
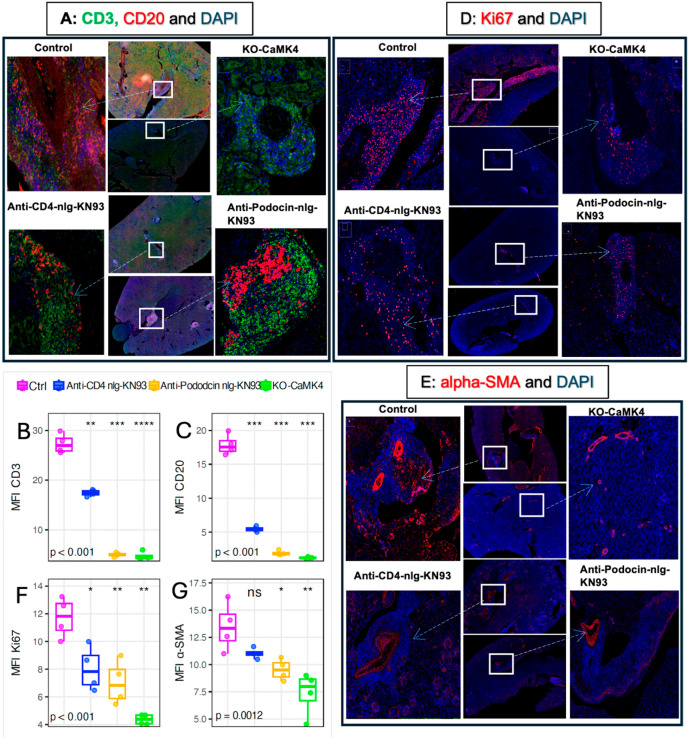
CaMK4 inhibition reduces formation and maturation of tertiary lymphoid structures (TLS) in kidneys of MRL/*lpr* mice. (**A**) Representative immunofluorescence staining shows organized TLS with T/B-cell segregation in controls and their disruption following CaMK4-targeted therapies. CD3 (green, T cells), CD20 (red, B cells), and DAPI (blue, nuclei). Scale bar = 20 µm. (**B**,**C**) Quantification of mean fluorescence intensity (MFI) for CD3 and CD20, in each treatment group. (n = 4 mice per group.) (**D**) Ki-67 (red) with DAPI shows proliferative activity within TLS, enriched in controls but markedly reduced in Camk4 knockout (KO) and nanogel-treated mice. White boxes indicate TLS regions. Scale bar = 20 µm. (**E**) α-SMA (red) with DAPI shows perivascular and interstitial fibroblast activation adjacent to TLS-rich areas, prominent in controls and reduced after CaMK4 inhibition. White boxes indicate TLS regions. Scale bar = 20 µm. (**F**,**G**) Quantification of mean fluorescence intensity (MFI) for KI-67 and a-SMA in each treatment group. (n = 4 mice per group); statistical comparisons were performed via one-way ANOVA with post hoc tests; (* *p*  <  0.05, ** *p*  <  0.01, *** *p*  <  0.001, **** *p* < 0.0001, ns means not significant) indicate which *p* values correspond to (**B**,**C**,**F**,**G**).

To evaluate adaptive immune components of TLS, we quantified CD3+ T cells and CD20+ B cells by dual immunofluorescence (Figure 3A). All treatment groups showed marked reductions in CD3^+^ and CD20^+^ mean fluorescence intensity (MFI) compared with controls (both *p* < 0.001), with the strongest suppression observed in CaMK4-deficient mice (Figure 3B,C). Among the pharmacologic arms, podocyte-targeted KN93 reduced CD3^+^ and CD20^+^ signals more than CD4-targeted delivery *(p*.adj < 0.001), indicating that CaMK4 inhibition in podocytes plays an important role in limiting lymphocyte recruitment to TLS.

### 2.3. Proliferation (Ki-67) and Stromal Activation (α-SMA)

To assess TLS maturation, we quantified Ki-67 (cellular proliferation) and α-SMA (stromal/myofibroblast activation) by immunofluorescence (Figure 3D,E). Compared with controls, CaMK4 deletion and CD4- or podocyte-targeted KN93 delivery significantly reduced Ki-67 and α-SMA expression (Figure 3F,G). Podocyte-targeted KN93 delivery produced a greater reduction in α-SMA expression than CD4-targeted delivery (*p* = 0.046; *p*.adj = 0.069), representing a borderline but biologically consistent difference. In contrast, Ki-67 levels did not differ between the two KN93 strategies (*p* = 0.41). These patterns parallel the observed reductions in T- and B-cell content, supporting a stronger suppression of TLS maturation and stromal remodeling with podocyte-directed CaMK4 inhibition.

## 3. Discussion

Our study provides the first comprehensive evidence that both genetic deficiency and cell-targeted pharmacologic inhibition of CaMK4 reshape the renal immune and stromal landscape in LN. Previous work has established a critical role for CaMK4 in T cells, by promoting Th17 differentiation and impairing Treg cell function, and in podocytes, by contributing to cytoskeletal disorganization and proteinuria [7]. Prior studies from our group have demonstrated increased CaMK4 expression in splenic T cells from diseased MRL/*lpr* mice, and targeted delivery of the CaMK4 inhibitor K93 to CD4 positive cells using nanoparticles limited kidney inflammation, suggesting that systemic immune activity precedes renal infiltration. We now demonstrate that CaMK4 also regulates the formation and expansion of TLS in LN.

CaMK4 inhibition significantly reduced TLS density and immune cell infiltration across all treatment groups of MRL/*lpr* mice, with the most pronounced effects observed in the podocyte-targeted arm. This is consistent with the concept that podocytes may contribute to immune cell recruitment through chemokines, such as CXCL13 [13]. Prior work [14] reported extensive, lymph node-like TLS networks in lupus-prone kidneys. In contrast, treated groups in our study exhibited only small, localized aggregates, primarily in the medulla and in areas adjacent to large blood vessels, indicating that CaMK4 suppression curtails TLS expansion, though it may not entirely prevent initial seeding. Because classical GC markers were not assessed and GC-like morphology was not consistently observed in this MRL/*lpr* material, we conservatively describe these infiltrates as TLS-like structure rather than mature GC-containing TLS [15,16]. The observed reduction in CD3^+^ T cells and CD20^+^ B cells across all treatment groups highlights the central role of CaMK4 in adaptive immune cell recruitment [17]. B cells are particularly critical for TLS maturation, and local autoantibody production [6]. Our results parallel the favorable outcomes achieved with B cell-targeted therapies, such as rituximab and belimumab in LN [18,19], but also emphasize the benefit of upstream, pathway-centered interventions.

Beyond immune infiltration, CaMK4 modulated downstream tissue responses critical to disease progression. In particular, analysis of Ki-67 and α-SMA expression highlighted proliferative and fibrotic components of renal pathology in LN [20,21]. CaMK4 inhibition led to a marked reduction in Ki-67 signal across all treatment groups, indicating a broad suppression of cellular proliferation within the kidney. This is consistent with previous reports linking elevated Ki-67 expression in human LN biopsies to active disease, high SLEDAI scores (SLE Activity Index), and poor renal outcomes [20]. Our results support the notion that CaMK4 drives proliferative responses in both immune and resident renal cells, thereby fueling TLS maturation and parenchymal damage. Similarly, α-SMA expression—used here as a marker of myofibroblast activation and fibrogenesis [22]—was significantly reduced, particularly in the podocyte-targeted group. While CD4^+^ T-cell-specific inhibition produced only modest reductions, podocyte-specific blockade of CaMK4 nearly abolished α-SMA expression, pointing to a dominant role of podocytes in fibrotic remodeling. These findings align with emerging evidence that injured podocytes release profibrotic mediators, including TGF-β, which drive interstitial matrix expansion and loss of kidney function [23].

Several limitations should be noted. TLS phenotyping was limited to CD3, CD20, Ki67, and α-SMA, and we did not assess canonical markers of TLS maturation (FDC, HEV, or germinal center markers). Because classical GC markers were not evaluated and GC-like morphology was not consistently observed in this MRL/*lpr* material [16], we describe these infiltrates as TLS-like aggregates rather than mature GC-containing TLS. Future studies will include CD21/CD35, PNAd, Bcl6/AID, and CXCL13 to stage TLS maturation where tissue availability permits. Because one group of animals involves germline Camk4 deficiency, developmental/compensatory effects cannot be fully excluded; however, the concordant results observed with postnatal, cell-targeted pharmacologic CaMK4 inhibition (and prior pharmacologic studies in lupus-prone mice) support a direct role for CaMK4 signaling in the renal phenotypes reported here. This study used the MRL/*lpr* (Fas-mutant) as a model of lupus nephritis. Because Fas deficiency can influence lymphocyte accumulation and the formation of lymphoid aggregates, some aspects of TLS development may be model-dependent. Future studies will validate these findings in additional LN models including NZB/W F1 lupus-prone mice. Lastly, all experiments were performed in female MRL/*lpr* mice, and it is self-evident that studies in male lupus-prone mice should assess whether CaMK4 inhibition or deletion controls the appearance of TLS-like aggregates in the kidney.

## 4. Materials and Methods

### 4.1. Animal Model and Experimental Design

Female MRL/l*pr* mice were housed under specific-pathogen-free conditions and randomized at 8 weeks into four groups (n = 4 per group): (1) wild-type controls receiving vehicle, (2) mice receiving CD4^+^ T-cell-targeted delivery of KN93, (3) mice receiving podocyte-targeted delivery of KN93 via anti-podocin–coated nanogels, and (4) *Camk4* knockout mice [9,12,24]. All mouse experiments were conducted in accordance with institutional guidelines and were approved by the Beth Israel Deaconess Medical Center Institutional Animal Care and Use Committee (BIDMC IACUC; Protocol #055-2024, “Pathogenesis and treatment of lupus”; approval date: 24 July 2025).

### 4.2. Histology, Inflammation Scoring and TLS Assessment

Kidneys were formalin-fixed, paraffin-embedded (FFPE), and stained with periodic acid–Schiff (PAS). Histologic sections were evaluated in a blinded manner. Interstitial inflammation was scored according to the Banff criteria, which assess the extent of mononuclear cell infiltration within the non-scarred cortical interstitium [25]. Because the Banff scoring system does not account for TLS, we additionally quantified TLS as discrete, organized lymphocytic aggregates (>50 cells) with or without germinal centers (GC). TLS density was calculated as the number of TLS per mm^2^ of renal tissue area [26] (Figure 1A).

### 4.3. Immunofluorescence Staining

FFPE kidney sections were stained as previously described [27]. Imaging was performed using a Keyence BZ-X800 microscope (Keyence Corporation, Osaka, Japan) (Figure 1B), and quantitative fluorescence was analyzed with ImageJ v1.53. To assess TLS features, sections were stained with antibodies against CD3 (T cells), CD20 (B cells), and Ki-67 (cell proliferation). α-SMA was included to evaluate stromal/fibroblast activation in TLS-rich areas. Full antibody details are provided in Supplementary Table S1A–C.

### 4.4. Statistical Analysis

All data are presented as mean ± SD. Comparisons among the four groups were performed using the Kruskal–Wallis exact test followed by Dunn’s post hoc test with Benjamini–Hochberg false discovery rate (BH/FDR) adjustment for multiple comparisons (reported as *p*.adj). Mann–Whitney U tests were used for pairwise comparisons where applicable. A two-tailed *p*-value < 0.05 was considered statistically significant. Analyses were conducted in R (v4.1.2).

## 5. Conclusions

Through cell-specific nanotherapies targeting CaMK4, we achieved coordinated suppression of TLS formation, local immune activation, and fibroblast activation, key hallmarks of LN progression. Notably, podocyte-targeted delivery of KN93 produced the most pronounced reduction in TLS-like structure, suggesting that podocyte-dependent pathways may contribute to interstitial inflammation and renal lymphoid neogenesis. We tried to introduce a mechanistic link between podocytes and renal lymphoid neogenesis, offering new insights into how TLS may originate and mature within the inflamed kidney. This spatial and cellular perspective identifies podocytes not only as passive targets of immune injury, but also as active participants in shaping the intrarenal immune microenvironment. Collectively, our findings establish CaMK4 as a central regulator of adaptive immunity, stromal remodeling, and podocyte injury in LN, and suggest that cell-targeted inhibition of this kinase may offer a strategy to disrupt local immune niches while minimizing systemic toxicity.

## Data Availability

The original contributions presented in this study are included in the article/Supplementary Material. Further inquiries can be directed to the corresponding author.

## Supplementary Materials

The following supporting information can be downloaded at: https://www.mdpi.com/article/10.3390/ijms27073190/s1.
