# Supplementary material for: Cell-Targeted Inhibition of CaMK4 Suppresses Tertiary Lymphoid-like Structure Development in Lupus-Prone Mice
Source: Int J Mol Sci. 2026 Mar 31;27(7):3190. doi: 10.3390/ijms27073190 (PMC13073123; doi:10.3390/ijms27073190)
Supplement: Supplementary file 1 [file ijms-27-03190-s001.zip › ijms-4166897-supplementary.pdf]

Table S1. Antibodies and materials used for immunofluorescence

A) Primary antibodies

Target	Vendor	Catalog no.	Notes
CD3	Bios	**BS-0765R**	FFPE; citrate Heat-induced epitope retrieval (HIER) (10 mM, pH 6.0); primary incubation **overnight, 4 °C**
CD20	Cell Signaling Technology	**98708**	Same conditions as above
Ki-67	eBioscience	**14-5698-82**	Same conditions as above
α-SMA	Abcam	**ab124964**	Same conditions as above

SMA=Smooth muscle actin

B) Secondary antibodies and nuclear stain

Reagent	Vendor	Catalog no.	Notes
Donkey anti-rabbit, Alexa Fluor–conjugated	Life Technologies	**A21206**	**1 h, RT**; matched to rabbit primaries
Goat anti-rabbit, Alexa Fluor–conjugated	Life Technologies	**A11011**	**1 h, RT**
Goat anti-rat, Alexa Fluor–conjugated	Life Technologies	**A11081**	**1 h, RT**
DAPI	Thermo Fisher	**S36973**	Nuclear counterstain, **1 h, RT**

C) Blocking and retrieval reagents (for completeness)

Reagent	Vendor	Catalog no.	Notes
Sodium citrate buffer, 10 mM (pH 6.0)	—	—	Heat-induced epitope retrieval
BSA 3%	Thermo Fisher	**BP1600-100**	Block, **1 h, RT**
Goat serum 5%	Thermo Fisher	**50-062Z**	Block alternative, **1 h, RT**
Donkey serum 5%	Sigma-Aldrich	**S30-100ML**	Block alternative, **1 h, RT**
